# Identification of a critical gene involved in the biosynthesis of the polyene macrolide lavencidin in *Streptomyces lavendulae* FRI-5 using the Target-AID (activation-induced cytidine deaminase) base editing technology

**DOI:** 10.1128/aem.00975-24

**Published:** 2025-04-22

**Authors:** Ryo Otsuka, Yu Sato, Kenji Okano, Eiji Okamura, Hiroya Tomita, Kohsuke Honda, Shigeru Kitani

**Affiliations:** 1 International Center for Biotechnology, Osaka University 13013, Suita, Osaka, Japan; 2 Research Center for Thermotolerant Microbial Resources, Yamaguchi University 13150, Yamaguchi, Japan; 3 Department of Life Science and Biotechnology, Faculty of Chemistry, Materials and Bioengineering, Kansai University 12860, Osaka, Japan; 4 Department of Chemistry and Biological Science, College of Science and Engineering, Aoyama Gakuin University 196857 , Sagamihara, Kanagawa, Japan; Washington University in St. Louis, St. Louis, Missouri, USA

**Keywords:** base editing, lavencidin, polyene macrolide antibiotics, *Streptomyces*, target-AID

## Abstract

Polyene macrolide antibiotics, produced mainly as secondary metabolites of streptomycetes, have distinct chemical structures and include clinically important antifungal drugs. We recently isolated the 28-membered polyene macrolide lavencidin from *Streptomyces lavendulae* FRI-5. Here, we identify and characterize the lavencidin biosynthetic (*lad*) gene cluster by combining a gene disruption system based on a base editing technology and *in silico* analysis. Sequence analysis of the draft genome of *S. lavendulae* FRI-5 revealed plausible lavencidin biosynthetic genes, which could be assigned roles in the biosynthesis of the polyketide backbone and the peripheral moiety, as well as in the regulation of lavencidin production. The introduction of a stop codon into the *ladA5* polyketide synthase (PKS) gene by the base editing system resulted in a complete loss of lavencidin production, indicating that the type I modular PKS system is responsible for the biosynthesis of lavencidin.

**IMPORTANCE:**

Polyene macrolide antibiotics display a unique mode of action among fungicides and exhibit potent fungicidal activity to which resistance does not readily develop. Deciphering the biosynthetic pathways of these fascinating compounds will provide chemical diversity for the development of industrially and clinically important agents. In this study, the Target-AID (activation-induced cytidine deaminase) system enabled us to identify the *lad* gene cluster involved in lavencidin biosynthesis, paving the way for the rational design of lavencidin derivatives with new or improved biological activity. Furthermore, this base editing system is capable of precisely and rapidly substituting the target nucleotide in several streptomycetes. Thus, our Target-AID system would be a powerful and versatile tool for the genetic engineering of streptomycetes as well as for analyzing the functions of uncharacterized genes, expanding the chemical diversity of useful bioactive compounds, and discovering novel natural products.

## INTRODUCTION

Secondary metabolites of microbial origin are structurally complex and prevalent in nature, providing a rich source of bioactive compounds that have been used in agriculture and human as well as veterinary medicine (1). Filamentous bacteria of the genus *Streptomyces* are the most promising microorganisms for the production of various bioactive compounds as secondary metabolites (2). Genome analysis, made possible by advances in DNA sequencing, has revealed that the large *Streptomyces* genome is rich in biosynthetic capacity, with approximately 20–40 biosynthetic gene clusters of secondary metabolites per strain (3). However, the functions of these biosynthetic genes and the entities of the secondary metabolites they encode remain to be elucidated (4). Therefore, the identification and characterization of the unexploited secondary metabolites and biosynthetic pathways would expand the chemical diversity of microbial bioactive compounds and lead to the discovery of novel enzymatic reactions.

**Fig 1:**
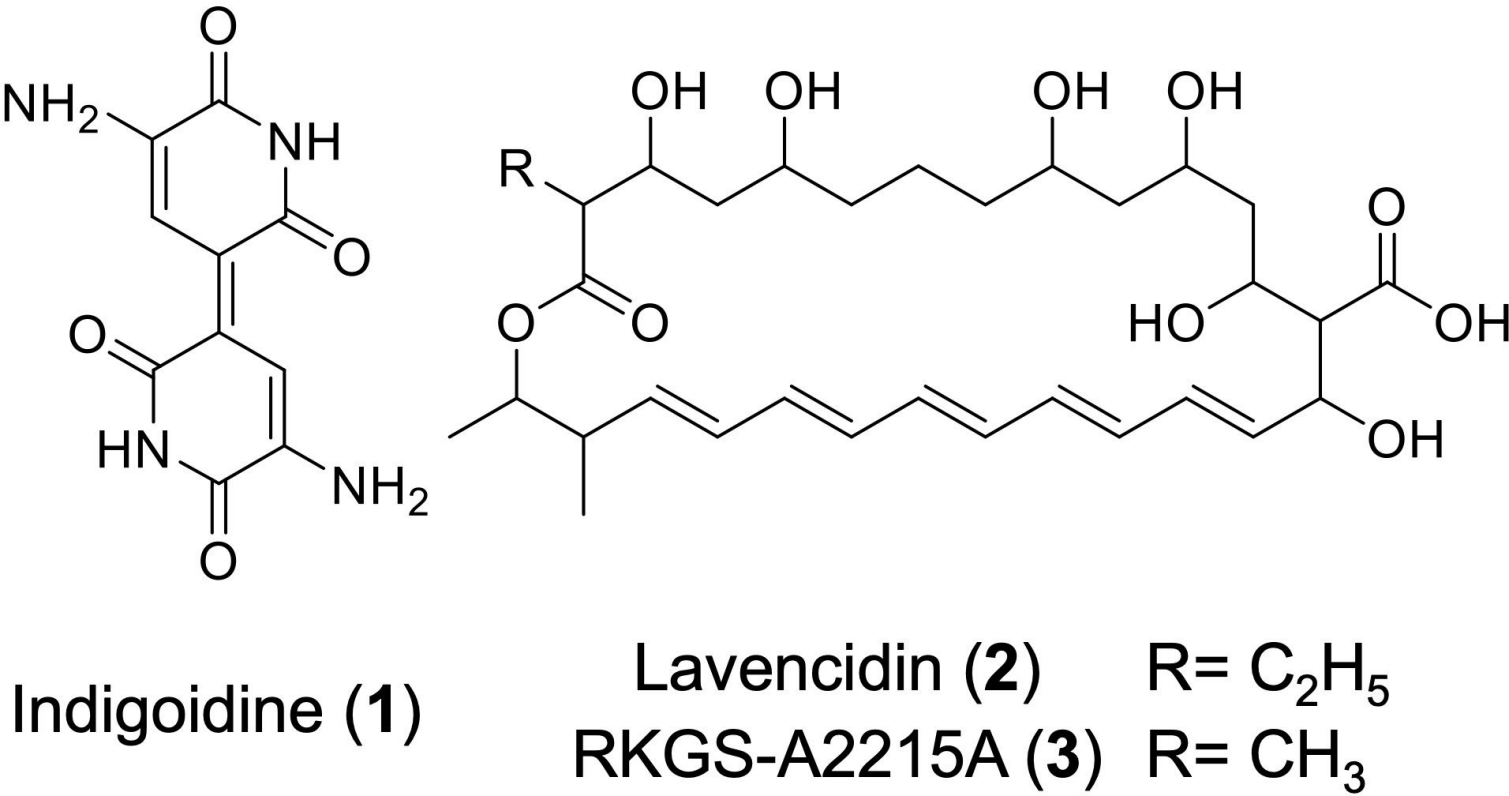
Chemical structures of indigoidine (**1**), lavencidin (**2**), and RKGS-A2215A (**3**) produced by *S. lavendulae* FRI-5.

*Streptomyces lavendulae* FRI-5 has a *Streptomyces* hormone, IM-2, which controls the production of four secondary metabolites (indigoidine [**1**, Fig. 1], a blue pigment; d-cycloserine, an antituberculosis agent; and showdomycin and minimycin, nucleoside antibiotics) (5, 6). In addition, this strain can synthesize the diol-containing polyketide lavendiol, which was isolated by genome mining and the heterologous expression of a cryptic biosynthetic gene cluster (7). We recently identified the new polyene macrolide antibiotic lavencidin (2), along with the known compound RKGS-A2215A (3), by using the OSMAC (one strain many compounds) approach (Fig. 1) (8, 9). These polyene macrolides contain a conjugated pentaene moiety together with six hydroxy groups and a carboxylic acid as a side chain. With respect to biological activity, lavencidin shows moderate growth-inhibitory activity against yeast and demonstrates cytotoxicity with low-micromolar IC_50_ values against human cancer cell lines. Interestingly, the cytotoxicity of lavencidin is weaker than that of RKGS-A2215A, in contrast to the antifungal activities detected from these compounds, suggesting that the biological activities of lavencidin and RKGS-A2215A depend on the length of the alkyl chain at C-2. Thus, elucidation of the biosynthetic pathway at the molecular level may generate lavencidin derivatives and facilitate the engineered biosynthesis of new antifungal and anticancer agents.

Programmable base editing without DNA cleavage has been developed based on CRISPR-Cas9 technology and is becoming a powerful tool for introducing mutations into genomic DNA (10). Notably, in cytosine base editors, the fusion protein of a cytidine deaminase and the nuclease-dead Cas9 (dCas9) recognizes the target DNA region at a specified sequence guided by the RNA and substitutes cytosine residue(s) to thymine residue(s) without inducing double-strand breaks. Meanwhile, it is much more difficult to genetically manipulate streptomycetes than model organisms such as *Escherichia coli* and *Saccharomyces cerevisiae*, partly because of the more diverse genomic content and extremely high GC content of streptomycetes (11–13). The analysis of gene function in streptomycetes has commonly involved homologous recombination-based gene disruption methods such as the PCR-targeting system (14–16). However, this traditional method of gene disruption is relatively time-consuming and labor-intensive, involving the construction of a cosmid DNA library and the preparation of a gene disruption vector, followed by multiple recombination steps. Recently, the Target-AID (activation-induced cytidine deaminase) system, which fuses the activation-induced cytidine deaminase ortholog (PmCDA1) from *Petromyzon marinus* with dCas9 or nickase Cas9 (nCas9), was established for cytidine base editing in the *Streptomyces* genome (17–19). However, only a few studies have utilized this base editing system for the functional identification of uncharacterized genes in nonmodel streptomycetes (20), as the efficacy of the system has primarily been demonstrated in the model streptomycete *Streptomyces coelicolor* A3(2).

In the present study, we identify a biosynthetic gene cluster for the polyene macrolide lavencidin by applying the Target-AID system in *S. lavendulae* FRI-5, and we propose a lavencidin assembly line with some unique biosynthetic features.

## RESULTS

### pLK101, a Target-AID vector for gene targeting

**Fig 2:**
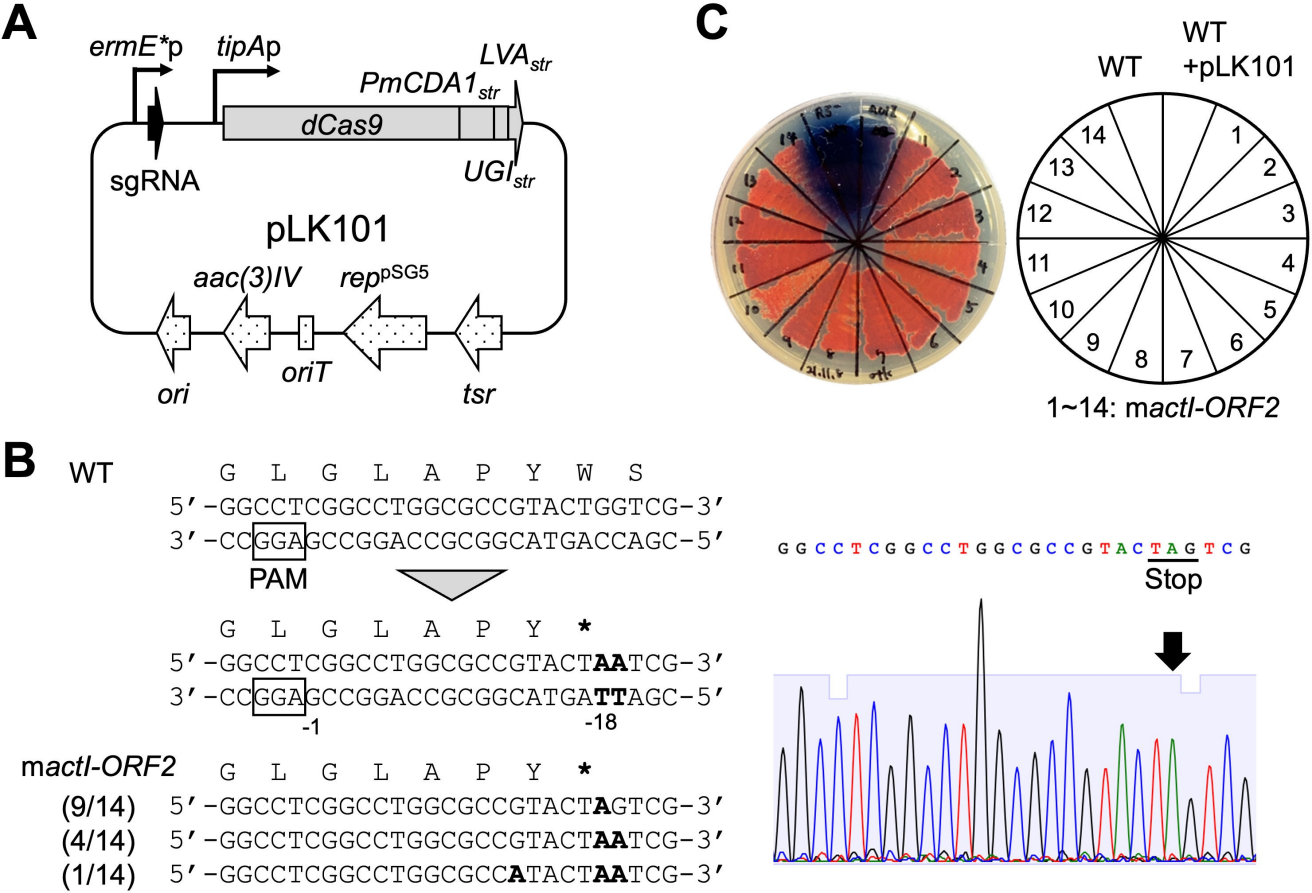
Target-AID base editing for *actI-ORF2* mutagenesis in *S. coelicolor* A3(2). (**A**) Map of the Target-AID vector pLK101. ermE*P, a strong constitutive promoter; sgRNA, gRNA scaffold with a 20-nt targeting sequence; *tipAp*, a thiostrepton-inducible promoter; *dCas9*, the nuclease dCas9 (D10A and H840A) gene; *PmCDA1_str_*, the cytidine deaminase PmCDA1 gene; *UGI_str_*, the UGI gene; *LVA_str_*, the protein degradation tag; *tsr*, a thiostrepton resistance gene; *rep^pSG5^*, a temperature-sensitive replication origin from pSG5; *oriT*, the origin of the transfer of plasmid RK2; *aac (3)IV*, an apramycin resistance gene; *ori*, high-copy-number origin of replication from ColE1. (**B**) Sequence alignment of the *actI-ORF2* locus edited by pTarget-ACT. The PAM sequence is shown in the box. The expected editing site is shown by the nucleotide number relative to the PAM sequence. The translated amino acid sequences are indicated above the corresponding nucleotide sequences. Mutated bases are shown in bold, and asterisks indicate stop codons. The numbers displayed to the left of the nucleotide sequence represent the ratio of the number of randomly selected exconjugants of stop codon-introduced clones to that of total sequenced clones. The right panel shows a representative Sanger sequencing chromatogram of the target region with the predicted C-to-T mutation (a mutated base is indicated by a black arrow). (**C**) Production of pigmented antibiotics in the m*actI-ORF2* strains. WT, the wild-type strain; WT + pLK101, the WT carrying pLK101; m*actI-ORF2*, m*actI-ORF2* strains. Each strain was streaked on R5^−^ agar medium and incubated for 4 days at 30°C. The plate was photographed from the bottom.

Several groups have developed Target-AID systems for genome editing in streptomycetes. These base editing systems have demonstrated that promoter activity for single guide RNA (sgRNA) expression is an important factor in the efficiency of genome editing (18, 19). Thus, we used the strong constitutive promoter ermE*P to express sgRNAs. In addition, the fused dCas9-CDA-UL_str_ protein was employed as a Target-AID‒derived base editor. This fusion protein includes dCas9, PmCDA1 (the cytidine deaminase), uracil DNA glycosylase inhibitor (UGI), and LVA (protein degradation tag) (17, 21, 22). The latter two proteins contribute to the improvement of mutational efficiency for the base editing of genes in streptomycetes (18). Moreover, to make it easier to express dCas9-CDA-UL, the fused region of CDA-UL was also codon-optimized for the expression of streptomycetes as in the case of the dCas9 protein, yielding dCas9-CDA-UL_str_. The expression of dCas9-CDA-UL_str_ is under the control of the thiostrepton-inducible *tipA* promoter (*tipA*p) (23). Finally, the base editing plasmid pLK101 (Fig. 2A) was constructed as described in Materials and Methods.

To validate our base editing system, we attempted to inactivate the *actI-ORF2* gene that encodes a polyketide chain-length factor involved in the biosynthesis of the blue-pigmented antibiotic actinorhodin (ACT) in the model streptomycete *S. coelicolor* A3(2), which also produces the red-pigmented antibiotic undecylprodigiosin (RED) (24). Inactivation of the *actI-ORF2* gene would result in the loss of ACT production, which could be easily identified by visual inspection. dCas9-CDA-UL_str_ has the ability to induce C-to-T editing within the −16 to −20 positions upstream of the protospacer adjacent motif (PAM) sequence when the designated guide sequence of sgRNA is 20 nt (18). Thus, we aimed to convert two cytosine residues (at the −18/−19 positions upstream of PAM) into thymine residues, which would result in the introduction of a stop codon (from TGG to TAG, TGA, and TAA) in the *actI-ORF2* gene (Fig. 2B) and prepared the base editing plasmid pTarget-ACT. *S. coelicolor* A3(2) carrying pTarget-ACT, designated as an m*actI-ORF2* strain, showed no production of blue pigment while remaining stable in producing red pigment, unlike the case of the wild-type strain carrying pLK101, which produced both blue and red pigments (Fig. 2C). This indicated that the introduction of pTarget-ACT leads to deficiency in ACT production, with no effect on RED production. Next, to confirm whether C-to-T editing was performed in the target region of the *actI-ORF2* gene—namely, whether a stop codon was generated—we analyzed the *actI-ORF2* sequences of the m*actI-ORF2* strains (Fig. 2B). The targeted cytosine residues were substituted to thymine residues in all 14 strains tested (Fig. S1). Unexpectedly, the base substitution at the −13 position upstream of the PAM sequence was observed in one strain. These results clearly indicated that the base editing plasmid pLK101 can induce the conversion of cytosine residues to thymine residues in the target sequence of *S. coelicolor* A3(2).

### Gene targeting with pLK101 in *S. lavendulae* FRI-5

**Fig 3:**
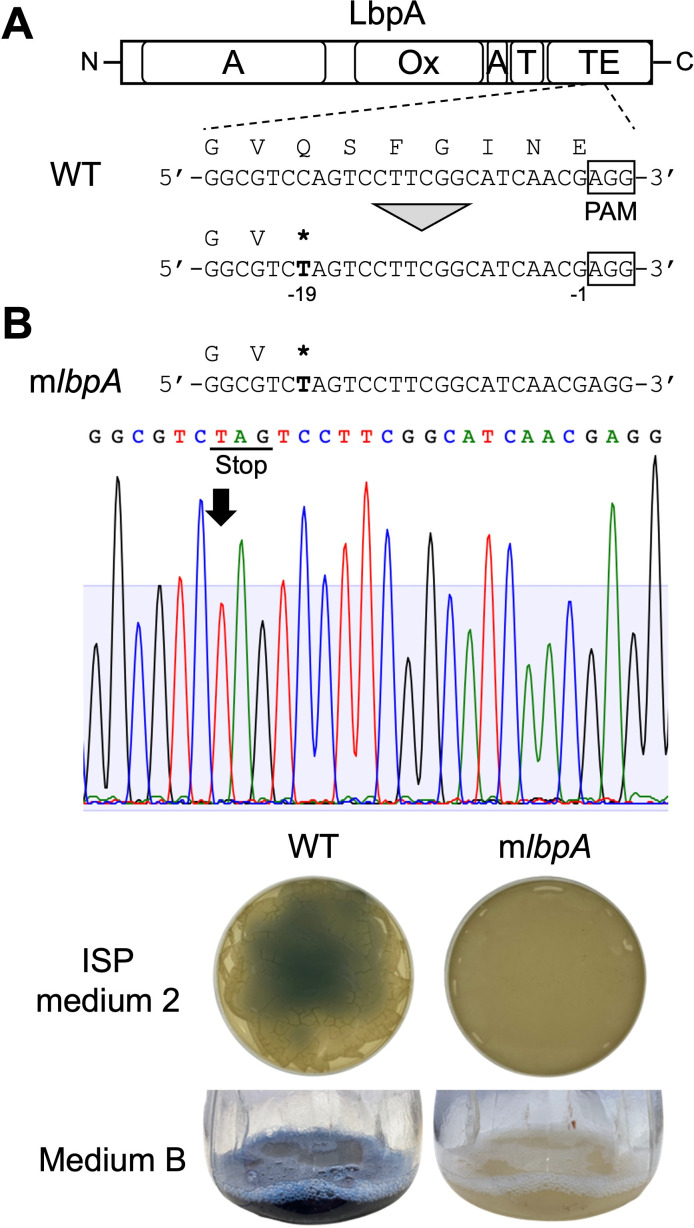
Indigoidine production in the m*lbpA* strain. WT, wild-type strain; m*lbpA*, m*lbpA* strains. The translated amino acid sequences are indicated above the corresponding nucleotide sequences. Mutated bases are shown in bold, and asterisks indicate stop codons. (**A**) Structural organization of LbpA and sequence alignment of the *lbpA* locus edited by pTarget-lbpA. The functional domains are shown: A, adenylation; Ox, oxidation; T, thiolation; TE, thioesterase. The PAM sequence is shown in the box. The expected editing site is shown by the nucleotide number relative to the PAM sequence. The translated amino acid sequences are indicated above the corresponding nucleotide sequences. A mutated base is shown in bold, and asterisks indicate stop codons. (**B**) Indigoidine production of the m*lbpA* strain grown on solid cultivation (top plate) and in liquid cultivation (bottom flask). The top panel shows a representative Sanger sequencing chromatogram of the target region with the predicted C-to-T mutation. A mutated base is indicated by a black arrow. For solid cultivation, each strain was cultivated on ISP medium two and incubated for 2 days at 28°C. The plate was photographed from the bottom. For liquid cultivation, each strain was inoculated into liquid medium B and incubated at 28°C for 7 h. IM-2-C_5_ was added at the final concentration of 1.2-ng/mL medium, followed by incubation for an additional 2 h.

The expression of dCas9-CDA-UL_str_ from pLK101 is driven by the thiostrepton-inducible *tipA*p. The *tipA*p requires the presence of the thiostrepton-responsive activator TipA belonging to the MerR-family transcriptional regulators, and the *tipA* gene is frequently encoded in the genomes of *Streptomyces* strains, including *S. coelicolor* A3(2) (25). Analysis of the genomic DNA sequence of *S. lavendulae* FRI-5 based on sequence homology to the *S. coelicolor* TipA (SCO3413) revealed a candidate gene encoding a putative TipA protein (SLA_TipA) with high similarity (92%) and identity (69%) to SCO3413 (Fig. S2). This finding suggests that the pLK101 plasmid is capable of editing the genome sequence of *S. lavendulae* FRI-5 as well as that of *S. coelicolor* A3(2). *S. lavendulae* FRI-5 produces the blue pigment indigoidine (**1**), which is synthesized by LbpA, a nonribosomal peptide synthetase (26). To investigate the possibility of pLK101 editing the genome sequence of *S. lavendulae* FRI-5, *lbpA* was selected as a target for gene editing because indigoidine production can be visually determined as in the case of *actI-ORF2* in *S. coelicolor* A3(2). For the inactivation of the LbpA function, sgRNA in the pTarget-lbpA was designed to convert a cytidine residue located at the −19 position upstream of the PAM sequence to a thymine residue. This conversion generated a TAG stop codon in the gene region encoding thioesterase (TE) domain of the LbpA protein (Fig. 3A). After pTarget-lbpA was introduced into *S. lavendulae* FRI-5 by intergeneric conjugation, the exconjugants were cultivated on the medium without apramycin for 7 days to cure the plasmid in order to avoid unexpected additional base substitutions. The loss of the plasmid in candidate strains was confirmed by PCR analysis. The expected mutation was verified by sequencing in the m*lbpA* strain, which could synthesize the truncated LbpA protein and is unlikely to produce indigoidine (Fig. 3B). On solid cultivation media, the production of the blue pigment indigoidine was not observed in the m*lbpA* strain, whereas the wild-type strain produced indigoidine (Fig. 3B). Even in liquid cultivation in the presence of IM-2, a *Streptomyces* hormone employed as an initiator of indigoidine biosynthesis, the m*lbpA* strain completely abolished the production of indigoidine production. Thus, the pLK101 plasmid has the ability to edit the genome sequence of *S. lavendulae* FRI-5 and would be useful for elucidating gene function in the organism’s physiology.

### Identification of the lavencidin biosynthetic gene cluster by gene targeting with pLK101

**Fig 4:**
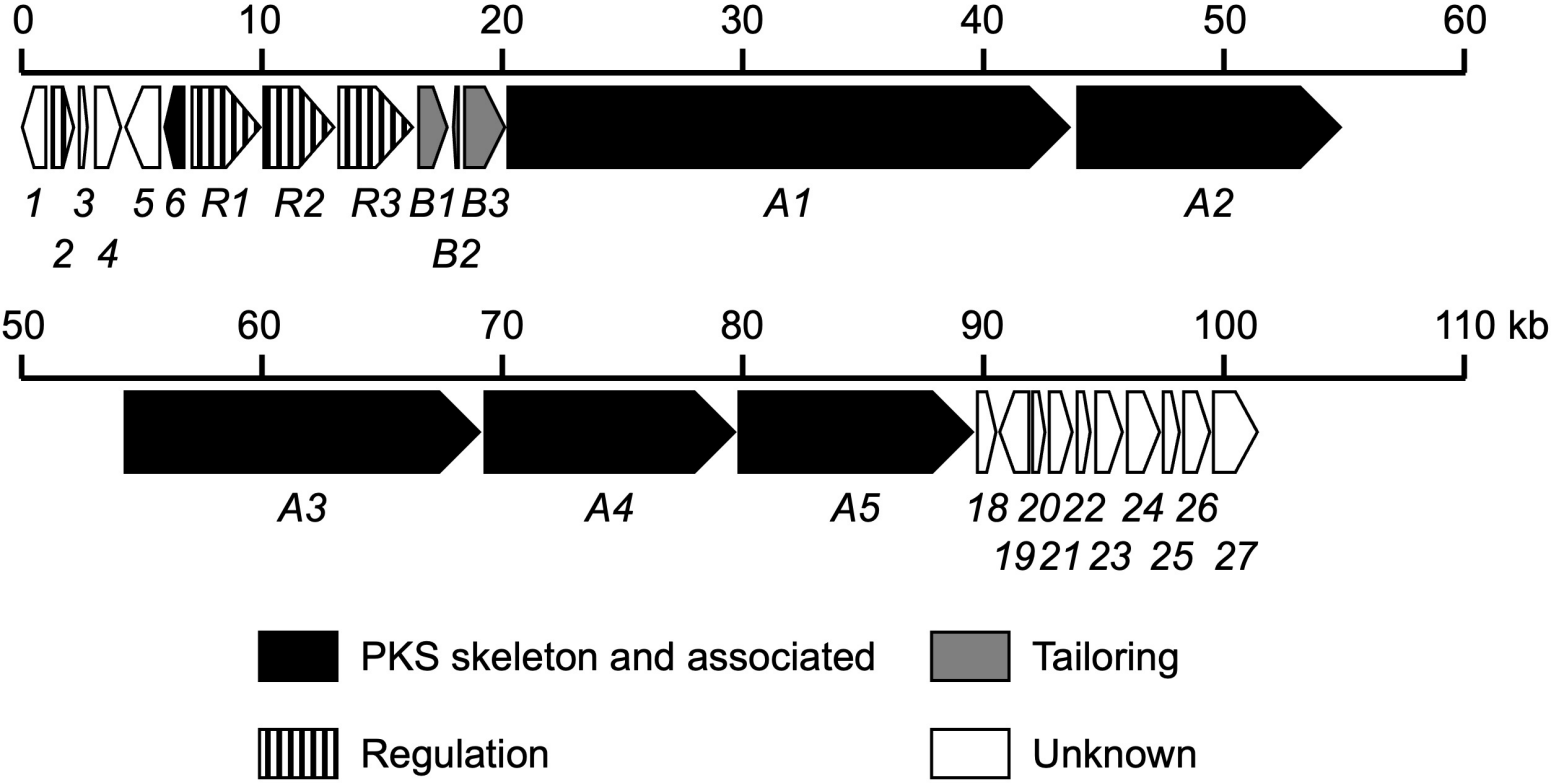
Genetic organization of the lavencidin biosynthetic gene cluster. Each arrow indicates the direction of transcription and the relative size of the gene. ORFs predicted to be involved in lavencidin biosynthesis are shaded. The proposed functions of each ORF are given here and summarized in Table 1.

To identify the lavencidin biosynthesis gene cluster in *S. lavendulae* FRI-5, we assumed that at least 62 enzymatic domains in the type I PKS modules are necessary for the lavencidin biosynthetic assembly line. As the genome sequence of *S. lavendulae* FRI-5 was decoded recently, we performed *in silico* screening of the draft DNA sequences using the antiSMASH tool, revealing a possible lavencidin biosynthetic gene cluster spanning 100.7 kb (Fig. 4). Annotation analysis of this region and comparison with the genes in the public databases resulted in 27 predicted open reading frames (ORFs). The genetic organization is shown in Fig. 4, and the deduced gene functions are summarized in Table 1.

**TABLE 1:** Deduced functions of ORFs in the lavencidin biosynthetic gene cluster

Gene	Size^*a*^	Proposed function	Homolog and origin^*b*^	Identity/similarity (%)
*orf1*	350	YncE-family protein	IPZ70_15500 (MCF3181332), *Streptomyces polychromogenes*	91/95
*orf2*	296	LysR-family transcriptional regulator	(WP_189530920), *Streptomyces roseolilacinus*	94/96
*orf3*	124	VOC-family protein	(WP_028801116), *Streptomyces* sp. 142MFCol3.1	92/96
*orf4*	356	GNAT-family *N-*acetyltransferase	IPZ70_15520 (MCF3181336), *Streptomyces polychromogenes*	86/90
*orf5*	465	Calcium-binding protein	PL81_39170(KIF00795), *Streptomyces* sp. RSD-27	87/92
*orf6*	268	4′-Phosphopantetheinyl transferase	IPZ70_15535 (MCF3181339), *Streptomyces polychromogenes*	85/88
*ladR1*	941	LuxR-family transcriptional regulator	(WP_161228434), *Streptomyces* sp. SID8352	81/85
*ladR2*	970	LuxR-family transcriptional regulator	(WP_078950727), *S. lavendulae*	67/72
*ladR3*	1019	LuxR-family transcriptional regulator	(WP_030241028), *S. lavendulae*	74/81
*ladB1*	398	Cytochrome P450	Svu004 (AFY08532), *Streptomyces virginiae*	100/100
*ladB2*	70	Ferredoxin	(WP_161228517), *Streptomyces* sp. SID8352	85/91
*ladB3*	542	GMC oxidoreductase	ChoL (ABS32193), *S. virginiae*	99/99
*ladA1*	7749	Type I polyketide synthase	(WP_167456781), *S. lavendulae*	77/82
*ladA2*	3636	Type I polyketide synthase	(WP_100660765), *S. lavendulae*	75/81
*ladA3*	5133	Type I polyketide synthase	(WP_100660764), *S. lavendulae*	78/84
*ladA4*	3767	Type I polyketide synthase	(WP_100660763), *S. lavendulae*	79/84
*ladA5*	3375	Type I polyketide synthase	(WP_100660762), *S. lavendulae*	79/84
*orf18*	257	Type II TE	(WP_045952057), *Streptomyces katrae*	91/96
*orf19*	397	Oxidoreductase	(WP_045952058), *Streptomyces katrae*	93/95
*orf20*	169	RidA-family protein	(WP_051840561), *S. lavendulae*	91/93
*orf21*	324	DUF4129 domain-containing protein	(WP_245691664), *Streptomyces katrae*	73/75
*orf22*	177	Hypothetical protein	GT031_31760 (MYV49999), *Streptomyces* sp. SID2888	74/79
*orf23*	367	Hypothetical protein	TNCT6_67860 (GED89701), *Streptomyces* sp. 6–11-2	94/96
*orf24*	447	DUF58 domain-containing protein	(WP_037661004), *Streptomyces griseofuscus*	84/90
*orf25*	220	Hypothetical protein	Hypothetical protein (WP_075662035), *Streptomyces acidiscabies*	75/80
*orf26*	353	Glycine/betaine ABC transporter ATP-binding protein	PL81_34430 (KIF01645), *Streptomyces* sp. RSD-27	96/97
*orf27*	598	Glycine/betaine ABC transporter permease	PL81_34425 (KIF01644), *Streptomyces* sp. RSD-27	91/94

^a^ Numbers refer to amino acid residues.

^*b*^ Parenthetical codes are National Center for Biotechnology Information accession numbers.

**Fig 5:**
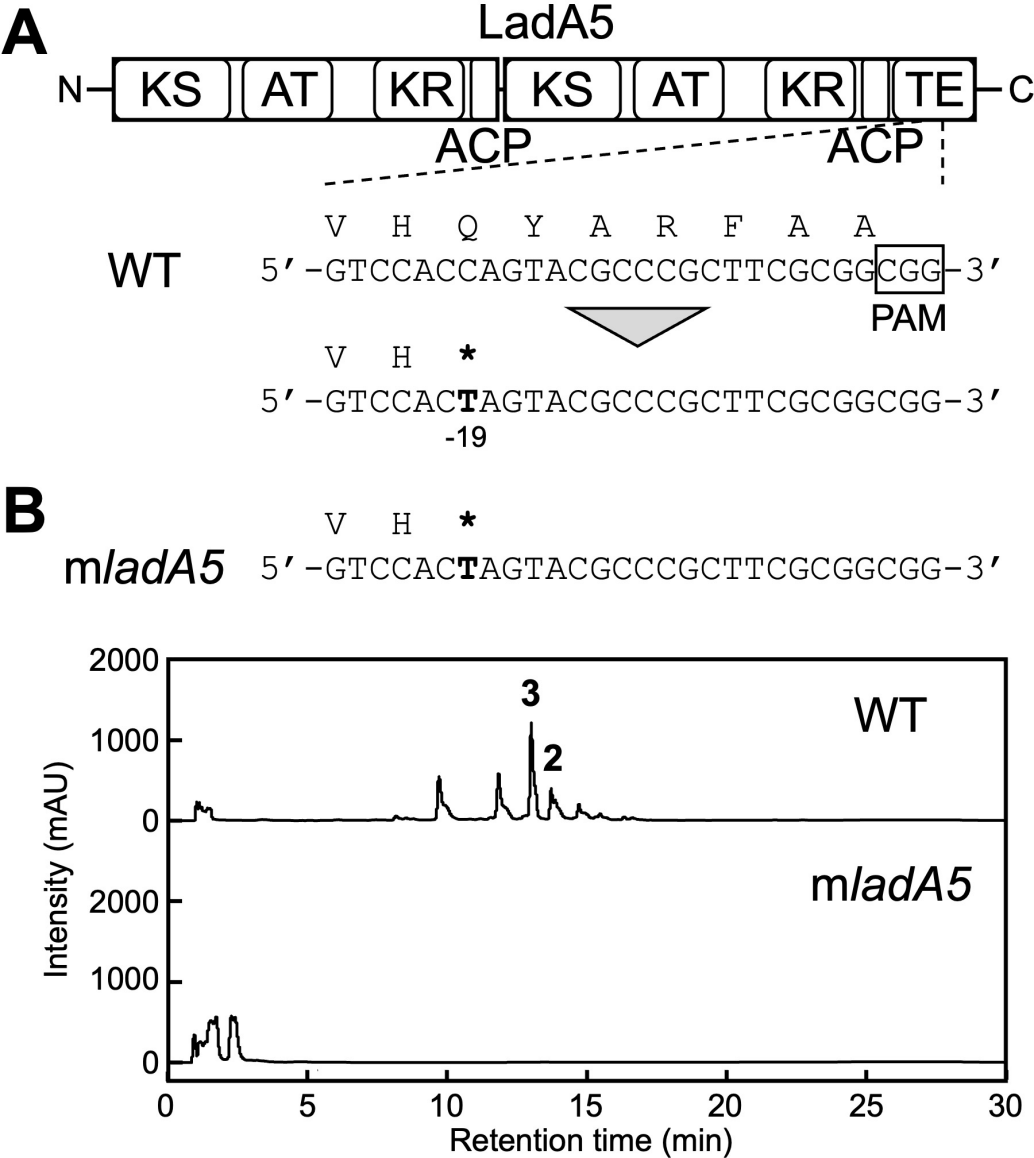
Lavencidin production in the m*ladA5* strain. WT, wild-type strain; m*ladA5*, m*ladA5* strain. The translated amino acid sequences are indicated above the corresponding nucleotide sequences. Mutated bases are shown in bold, and asterisks indicate stop codons. (**A**) Structural organization of LadA5 and sequence alignment of the *ladA5* locus edited by pTarget-ladA5. The functional domains are shown: KS, ketosynthase; AT, acyltransferase; KR, ketoreductase; ACP, acyl carrier protein; TE, thioesterase. The PAM sequence is indicated by the black box, and the expected editing site is shown by the nucleotide number relative to the PAM sequence. (**B**) HPLC chromatograms of n-butanol extracts for analysis of lavencidin production. mAU, milliabsorbance units at 290 nm. Lavencidin (**2**) and RKGS-A2215A (**3**) were eluted at 13.7 and 13.0 min, respectively.

Five PKS genes (*ladA1*, *ladA2*, *ladA3*, *ladA4*, and *ladA5*) were identified in the cluster and oriented in the same direction, together with *ladB* genes, which might be involved in the modification process of polyketide biosynthesis, and *ladR* genes, which might control the production of lavencidin. These clustered regulatory genes are often found in the biosynthetic gene clusters of polyene macrolides (27–30). The *ladA5* gene, which encodes only one TE domain in the PKS, was inactivated by introducing a stop codon into the region encoding the TE domain of LadA5 using pLK101 (Fig. 5A), and the desired strain was named the m*ladA5* strain (Fig. 5B). The m*ladA5* strain was unable to produce lavencidin (2) and RKGS-A2215A (3) along with several compounds (Fig. 5B). This result indicated that *ladA5*, a PKS gene, contributed to the polyketide assembly of **2** and **3**, and implied that other *ladA* PKS genes, probably organized in a polycistronic operon with *ladA5*, are also involved in the biosynthesis of these polyene macrolides. Thus, we concluded that the *lad* cluster, consisting of the *ladA*, *ladB*, and *ladR* genes, is responsible for the production of lavencidin and RKGS-A2215A with their derivative compound(s).

### *In silico* analysis of the gene cluster for lavencidin biosynthesis

**Fig 6:**
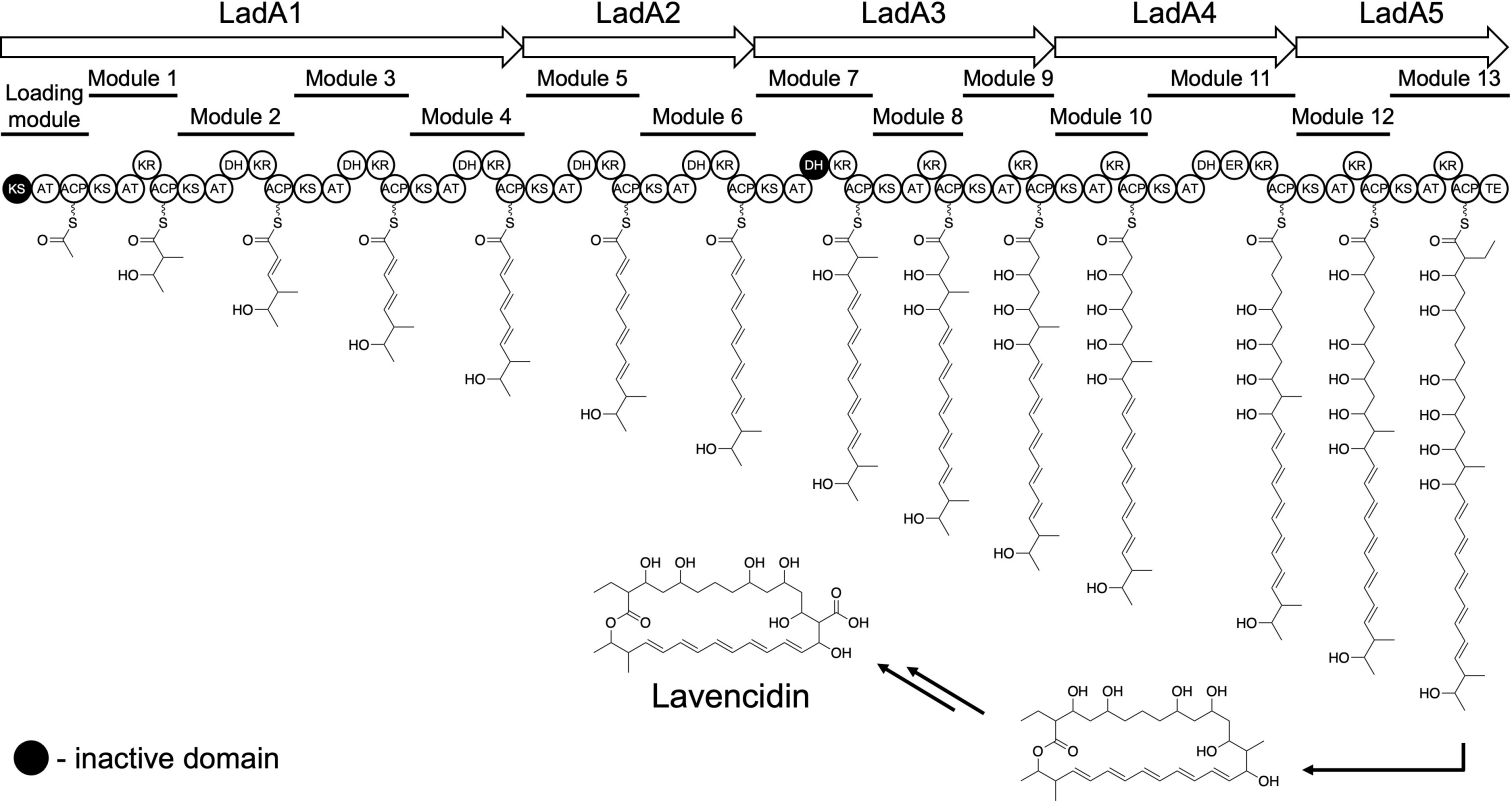
Predicted model for lavencidin biosynthesis. The circles represent enzymatic domains in the PKS polypeptide: KS, ketosynthase; AT, acyltransferase; KR, ketoreductase; ACP, acyl carrier protein; DH, dehydratase; ER, enoylreductase; TE, thioesterase. The presumed inactive KS and DH domains of the loading module and module seven are shaded in black.

To predict the polyketide assembly process in the lavencidin biosynthetic pathway, five PKSs (LadA1 to LadA5) were analyzed *in silico*, demonstrating that a total of 64 enzymatic domains are organized into 14 PKS modules (Fig. 6). The number of domains predicted here is two more than we had initially expected. The PKS module in the typical type I PKS machinery has a minimal domain set consisting of acyltransferase (AT), ketosynthase (KS), and acyl carrier protein (ACP) domains (31). With respect to the KS domains from LadA PKSs, analysis of the conserved active site (the catalytic triad of Cys-His-His) revealed that the KS domain in the loading module (KSL) has no corresponding amino acids and lacks a significant conserved motif (Fig. S3) (32), suggesting a high possibility that KSL has lost its original function as a KS. Conversely, other KS domains (KS1 to KS13) have the conserved active site, indicating that they all function in the condensation steps.

Based on phylogenetic tree analysis and sequence alignment of the conserved functional motif of the AT domain within each module (Fig. S4 ; Fig. S5 ), we predicted the substrate specificities of the AT domains. Our analysis demonstrated that the AT domains of modules 2, 3, 4, 5, 6, 8, 9, 10, 11, and 12 are specific for malonyl-CoA. In contrast, the AT domains of the loading module and modules 1, 7, and 13 are located evolutionarily close to other AT domains that recognize methylmalonyl-CoA as a substrate. However, the position of LadA1_ATL in the phylogenetic tree is slightly different from the positions of AT1, AT7, and AT13, and some of the amino acids around the conserved motif differ among ATL, AT1, AT7, and AT13, suggesting the possibility that ATL recognizes other substrates (e.g., acetyl-CoA) rather than methylmalonyl-CoA. These predicted substrates are nearly identical to the carbon skeleton of RKGS-A2215A (3), indicating that the *lad* cluster is responsible for the biosynthesis of at least RKGS-A2215A. With respect to ACP domains, all 14 ACP domains are presumed to be functional, as they each have a signature motif around the Ser residue as a 4′-phosphopantetheine attachment site (Fig. S6) (33).

With respect to the KR domains, all 13 KR domains contain the consensus sequence for the NADP(H)-binding site and a typical catalytic triad (Ser-Tyr-Asn) (Fig. S7) (34), indicating that all of the KR domains should be active in the polyketide assembly line. All seven of the predicted dehydratase (DH) domains contain the conserved His residue in the conserved HXXXGXXXXP motif and two active sites (Asp and His residues) (Fig. S8) (35), suggesting that these DH domains appear to be responsible for the formation of a double bond. However, the polyketide biosynthesis of lavencidin and RKGS-A2215A does not require the DH activity of module 7, suggesting that DH7 is probably inactive in the assembly line. Similar discrepancies have been found in the biosynthetic pathways of other polyketides, such as nystatin and rifamycin (29, 36). The polyketide assembly line has the only enoylreductase (ER) domain that is found in module 11 of LadA4. ER11 contains the conserved NADP(H)-binding site (Fig. S9) and is therefore likely responsible for the reduction of a double bond between C-7 and C-8 (37). Based on the predicted LadA PKS function, we propose that the biosynthetic mechanism of lavencidin initiates the recognition of acetyl-CoA by the ACP domain of the loading module (Fig. 6).

## DISCUSSION

Polyene macrolide antibiotics have a distinct chemical structure that includes multiple conjugated double bonds and multiple hydroxy groups on the polyketide macrolide ring. This type of antibiotic binds specifically to ergosterol in filamentous fungi and yeasts, forming a complex that alters the structure of the cell membrane and kills these microorganisms (38, 39). Thus, elucidating the biosynthetic pathways of polyene macrolide antibiotics could lead to an expansion of their chemical diversity with enhanced antifungal activity through genetic engineering. We recently found that lavencidin, a new member of the 28-membered ring polyene macrolide antibiotics, is a secondary metabolite of *S. lavendulae* FRI-5 (9). In the present study, we successfully identified the biosynthetic gene cluster of lavencidin by genome editing using the newly developed Target-AID vector pLK101 and proposed a biosynthetic model consisting of a polyketide assembly line. Moreover, this base editing system would facilitate efficient gene functional analysis in both the model strain *S. coelicolor* A3(2) and the unexplored strain *S. lavendulae* FRI-5.

The biosynthetic mechanism of the polyketide core structure of lavencidin can mostly be explained by the assembly line of the canonical type I PKS system. However, the initiation process of the lavencidin assembly line appears to be an intriguing step for the incorporation of acetyl-CoA onto the loading module. Significant differences in the conserved motif between LadA1_KSL and other KS domains (Fig. S3) suggest that LadA1_KSL probably does not have an enzymatic function, such as that of decarboxylase for synthesizing an acetyl group from malonate and that of KS for a Claisen condensation. On the other hand, in the phylogenetic tree, LadA1_ATL is positioned in the clade of the AT domains that recognize methylmalonyl-CoA as a substrate but at a slightly greater evolutionary distance (Fig. S4). In addition, it is interesting to note that LadA1_ATL possesses all the conserved amino acids essential for AT activity, whereas the amino acid sequence surrounding these conserved residues differs from those of other AT domains (Fig. S5). These observations implied the possibility that LadA1_ATL is specific for substrates other than methylmalonyl-CoA or that the LadA1_ATL function is not active. A putative discrete enzyme (Orf4), a member of the GNAT-family *N*-acetyltransferase, is present in the downstream region of *orf6*, which encodes 4′-phosphopantetheinyl transferase. A GNAT domain of the loading module in curacin A biosynthesis catalyzes the loading of an acetyl group onto the ACP domain (40), suggesting that Orf4 might be involved in the initial step of lavencidin biosynthesis.

After the LadA PKS assembly line synthesizes the polyene macrolide core structure, a modification process to form a carboxylic group at C-14 is essential for the biosynthesis of lavencidin. In the rosamicin biosynthetic pathway, the cytochrome P450 enzyme RosC converts the methyl group of 20-deoxo-20-dihydrorosamicin to the carboxylic group for the production of 20-carboxyrosamicin via the hydroxylation, formylation, and carboxylation steps (41). Among the biosynthetic genes of lavencidin, *ladB1* encodes a putative cytochrome P450 protein, while *ladB2* and *ladB3* encode products that are thought to mediate electron transfer in the P450 catalytic cycle. Thus, we postulate that these LadB proteins are tailoring enzymes responsible for the formation of the C-14 carboxylic group of lavencidin.

Over the past decade, several genome editing tools have been developed in streptomycetes (18, 19, 42–44). The Target-AID base editing system, which can edit target genes with high efficiency, is one of the next-generation genome editing tools. However, few reports have used this promising technique to analyze the functions of uncharacterized genes (20). Using pWHU77-BE, a different type of Target-AID vector from pLK101 utilized in this study, the biosynthetic genes of hygromycin B have been identified. Our Target-AID vector pLK101 is also capable of inactivating target genes in the unexplored strain *S. lavendulae* FRI-5 as well as in the model streptomycete *S. coelicolor* A3(2). Furthermore, the pLK101 vector enabled the identification of uncharacterized biosynthetic genes for the attractive polyene macrolide antibiotic lavencidin. The genome of *S. lavendulae* FRI-5 possibly harbors at least 38 clusters of genes involved in the biosynthesis of secondary metabolites, whereas only three biosynthetic gene clusters have been identified for lavencidin, indigoidine, and lavendiol thus far. Target-AID base editing technology allows us to substitute an amino acid in the target protein in addition to introducing the stop codon into the target gene. In addition, the discovery of cryptic compounds will be facilitated by this technology, which impairs the function of negative regulators for secondary metabolite production or modifies the promoter sequence to increase the expression levels of silent genes. Thus, the Target-AID vector pLK101 could be a valuable tool for the discovery of novel natural products from streptomycetes.

## MATERIALS AND METHODS

### Bacterial strains, plasmids, and growth conditions

For sporulation, *S. coelicolor* A3(2) M145 was grown at 30°C on mannitol soya flour (MS) medium (14), and *S. lavendulae* FRI-5 (MAFF10-06015; National Food Research Institute, Tsukuba, Japan) was grown at 28°C on ISP medium 2 (Becton, Dickinson and Company, Franklin Lakes, NJ, USA). *E. coli* DH5α was used for general DNA manipulation, and the DNA methylation-deficient *E. coli* strain ET12567 containing the RP4 derivative pUZ8002 was used for *E. coli*/*Streptomyces* conjugation (45). *E. coli* strains were grown in Luria‒Bertani medium supplemented with appropriate antibiotics as required. The general *E. coli* and *Streptomyces* manipulations were performed as described previously (14). pCRISPR-dCas9 was a gift from Tilmann Weber (Addgene plasmid #125687; https://www.addgene.org/125687/; RRID:Addgene_125687) and was used to construct the gene inactivation plasmid. The primers used in this work are listed in Table S1.

### Construction of Target-AID vectors

The cytidine deaminase gene (*PmCDA1*) from *P. marinus*, the uracil-DNA glycosylase inhibitor (UGI) gene, and the protein degradation tag (LVA) were codon-optimized for streptomycetes and synthesized as a fused gene (*CDA-UL_str_*) by GeneArt Gene Synthesis (Thermo Fisher Scientific, Waltham, MA, USA). The fused gene, which also contains a region for several linker peptides (a GS linker, SH3 domain, and 3 × FLAG tag) in the upstream region of *PmCDA1*, was cloned into the pMK-RQ vector (Thermo Fisher Scientific), generating pMQ-CDA-UL*_str_*. The DNA sequences of codon-optimized genes used in this study are listed in Table S1. The fused gene was PCR-amplified using pMQ-CDA-UL*_str_* as a template and the primer pair PmCDA1-Fw/PmCDA1-Re. Meanwhile, a 4.0-kb PCR fragment of *dCas9* was amplified from pCRISPR-dCas9 using the primer pair dCas9-Fw/dCas9-Re. The two resulting segments were cloned into the *Nde*I and *Hin*dIII sites of pCRISPR-dCas9 using NEBuilder HiFi DNA Assembly (New England Biolabs, Ipswich, MA, USA) to yield pLK101. The plasmid pLK101 was digested with *Nco*I and ligated with single-stranded DNA oligonucleotides (ssDNA oligos) for gene targeting by Gibson assembly with the following protocol (https://www.neb.com/en/protocols/2022/03/04/protocol-for-bridging-double-stranded-dna-with-a-single-stranded-dna-oligo-using-nebuilder-hifi-dna-assembly). The linearized pLK101 was bridged by ssDNA oligos, resulting in the generation of the desired gene targeting plasmid. The ssDNA oligos were designed as 5′-CGGTTGGTAGGATCGACGGC-N20-GTTTTAGAGCTAGAAATAGC-3′. ssDNA-actI-ORF2, ssDNA-lbpA, and ssDNA-ladA5 were used as ssDNA oligos to inactivate the *actI-ORF2*, *lbpA*, and *ladA5* genes, respectively. The resulting plasmids were designated as pTarget-ACT, pTarget-lbpA, and pTarget-ladA5*,* respectively.

### Construction of the *S. coelicolor actI-ORF2* mutant and analysis of actinorhodin production

*E. coli* ET12567(pUZ8002) harboring pTarget-ACT was conjugated with *S. coelicolor* A3(2). The introduction of the plasmid pTarget-ACT was confirmed through apramycin resistance (50 µg/mL) measurements and PCR analysis. Exconjugants were grown for 5 days on medium containing both thiostrepton (20 µg/ml) to facilitate expression of the dCas9-cytidine deaminase fusion protein and apramycin for plasmid retention. The genotype of candidate strains for the *actI-ORF2* mutation was confirmed by DNA sequencing, and the *S. coelicolor actI-ORF2* mutant was designated as the m*actI-ORF2* strain. The m*actI-ORF2* strain was grown on R5− agar medium (46) and incubated at 30°C for 4 days to analyze actinorhodin production.

### Construction of the *lbpA* and *ladA5* mutants in *S. lavendulae* FRI-5

For the inactivation of *lbpA*, pTarget-lbpA was introduced into *S. lavendulae* FRI-5 by intergeneric conjugation. Mutant strains of *lbpA* were obtained in the same way as for the construction of the m*actI-ORF2* strain, except that the thiostrepton concentration (5 µg/mL) for the expression of the dCas9-cytidine deaminase fusion protein and the cultivation condition (37°C for 7 days) for the curing of the plasmid were different. To confirm the loss of pTarget-lbpA in the strains, a 2.1-kb fragment of *dCas9* and *PmCDA1* genes was analyzed by PCR with the primer pair cPCR-check-Fw (5′- GCAAGGACTTCCAGTTCTACAAGGT-3′)/cPCR-check-Rv (5′- GCTTCAGGGTCTTCTCGAGCCA-3′). The genotype of candidate strains for the *lbpA* mutation was confirmed by DNA sequencing, and the *S. lavendulae lbpA* mutant was designated the m*lbpA* strain.

For the search for lavencidin biosynthetic genes, the genomic DNA of *S. lavendulae* FRI-5 was sequenced using the PacBio RS II platform (Pacific Biosciences, Menlo Park, CA, USA). A *de novo* assembly was performed using the Hierarchical Genome Assembly Process ver. 3.0 (HGAP3) within single-molecule real-time analysis software based on a Celera assembler, resulting in three contigs. The assembled contig sequences were used to screen the genome for putative lavencidin biosynthetic genes. ORFs and gene functions were manually annotated using the antiSMASH program (https://antismash.secondarymetabolites.org/). To inactivate *ladA5*, pTarget-ladA5 was constructed using the genomic information described above. Mutant strains of *ladA5* were obtained in the same way as for the construction of the m*lbpA* strain. The genotype of candidate strains for the *ladA5* mutation was confirmed by DNA sequencing, and the *S. lavendulae ladA5* mutant was designated the m*ladA5* strain.

### Analysis of indigoidine and lavencidin production in the *S. lavendulae* mutants

For the analysis of indigoidine production on solid medium, spores (1.0 × 10^8^ CFU) of the *S. lavendulae* FRI-5 strains were inoculated on ISP medium two and incubated at 28°C for 2 days. For liquid cultivation, these strains were cultivated as described previously. After 5 h of cultivation, IM-2-C_5_ was added to culture broth at a final concentration of 1.2-µg/mL medium (26). The culture supernatant was collected and filtered through a 0.2-µm pore size filter, and the absorbance at 590 nm was measured to quantify indigoidine production. For the analysis of lavencidin production, 1 mL of the seed culture of the m*ladA5* strain was inoculated into 70 mL of A-2M medium (containing [in grams per liter] soluble starch, 20; soya flour, 15; yeast extract, 2; and CaCO_3_, 4 [pH 6.2]) in a 500-mL baffled flask and cultured at 160 rpm and 28°C (9). After 24 h of cultivation, the culture broth was extracted with a half volume of n-butanol. After evaporation of the organic layer and dissolution with DMSO, the crude extract was subjected to reversed-phase high-performance liquid chromatography (HPLC) on a Cadenza CD-C_18_ column (3 µm; 4.6 × 250 mm; Imtakt, Kyoto, Japan) developed with a gradient system of CH_3_CN (15% for 0–3 min; 15%–85% for 3–25 min; 85% for 25–29 min) containing 0.1% formic acid (flow rate, 1.2 mL/min; UV detection, 290 nm).

## Data Availability

The nucleotide sequence data reported in this paper have been deposited in NCBI/ENA/DDBJ under accession number LC815377.

## SUPPLEMENTAL MATERIAL

Supplemental material aem.00975-24-s0001.pdf Tables S1 and S2; Figures S1 to S9.

ASM does not own the copyrights to Supplemental Material that may be linked to, or accessed through, an article. The authors have granted ASM a non-exclusive, world-wide license to publish the Supplemental Material files. Please contact the corresponding author directly for reuse.
