# Supplementary material for: Identification of a critical gene involved in the biosynthesis of the polyene macrolide lavencidin in Streptomyces lavendulae FRI-5 using the Target-AID (activation-induced cytidine deaminase) base editing technology
Source: Appl Environ Microbiol. 2025 Apr 22;91(5):e00975-24. doi: 10.1128/aem.00975-24 (PMC12093942; doi:10.1128/aem.00975-24)
Supplement: Supplemental material — Tables S1 and S2; Figures S1 to S9. [file aem.00975-24-s0001.pdf]

Table S1. Oligonucleotides used in this study

Primer	Sequence (5' – 3')
dCas9-Fw	ACGGCGTCAGAGAAGGGAGCGGACA
dCas9-Re	GAGCCGCCGCCGCCGTCGCCGCCGAG
PmCDA1-Fw	CTCGGCGGCGACGGCGGCGGCGGCTC
PmCDA1-Re	TACCCGGGGATCCTCTAGAAAGCTTTCAGGCGACGAGCAG
ssDNA-actI-ORF2	CGGTTGGTAGGATCGACGGCACCAGTACGGCGCCAGGCCGGTTTTAGAG CTAGAAATAGA
ssDNA-lbpA	CGGTTGGTAGGATCGACGGCCCAGTCCTTCGGCATCAACGGTTTTAGAG CTAGAAATAGA
ssDNA-ladA5	CGGTTGGTAGGATCGACGGCCCAGTACGCCCGCTTCGCGGGTTTTAGAG CTAGAAATAGC

Table S2. DNA sequences of codon-optimized genes for the construction of the pLK101 vector

Name	Sequence
PmCDA1$_{str}$	ATGACCGACGCCGAGTACGTCCGCATCCACGAGAAGCTGGACATCTACA<br>CCTTCAAGAAGCAGTTCTTCAACAACAAGAAGTCCGTCAGCCACCGCTG<br>CTACGTCCTGTTCGAGCTGAAGCGCCGGGGCGAGCGCCGCGCCTGCTTC<br>TGGGGCTACGCCGTCAACAAGCCGCAGTCCGGCACCGAGCGGGGCATCC<br>ACGCCGAGATCTTCTCCATCCGCAAGGTCGAGGAGTACCTGCGCGACAA<br>CCCGGGCCAGTTCACGATCAACTGGTACTCCAGCTGGTCCCCGTGCGCC<br>GACTGCGCCGAGAAGATCCTCGAGTGGTACAACCAGGAGCTGCGCGGCA<br>ACGGCCACACGCTGAAGATCTGGGCCTGCAAGCTGTACTACGAGAAGAA<br>CGCCCGCAACCAGATCGGCCTGTGGAACCTCCGCGACAACGGCGTCGGC<br>CTGAACGTCATGGTGTCCGAGCACTACCAGTGCTGCCGCAAGATCTTCA<br>TCCAGTCCTCGCACAACCAGCTGAACGAGAACCGCTGGCTCGAGAAGAC<br>CCTGAAGCGGGCCGAGAAGCGCCGCTCCGAGCTGTCCATCATGATCCAG<br>GTCAAGATCCTGCACACCACCAAGTCGCCGGCCGTC
UGI$_{str}$	ATGACCAACCTGTCCGACATCATCGAGAAGGAGACCGGCAAGCAGCTGG<br>TCATCCAGGAGTCCATCCTGATGCTCCCCGAGGAGGTCGAGGAGGTCAT<br>CGGCAACAAGCCCGAGTCCGACATCCTGGTCCACACCGCCTACGACGAG<br>TCCACCGACGAGAACGTGATGCTGCTGACCTCCGACGCGCCCGAGTACA<br>AGCCGTGGGCGCTCGTGATCCAGGACTCCAACGGCGAGAACAAGATCAA<br>GATGCTG
LVA$_{str}$	CTCGTCGCC
Linker region (GS linker, SH3 domain and 3×FLAG tag)	GGCGGCGGCGGCTCCGGCGGCGGCGGCAGCGCCGAGTACGTGCGCGCCC<br>TGTTCGACTTCAACGGCAACGACGAGGAGGACCTGCCGTTCAAGAAGGG<br>CGACATCCTGCGCATCCGCGACAAGCCCGAGGAGCAGTGGTGGAACGCC<br>GAGGACTCCGAGGGCAAGCGCGGCATGATCCCGGTGCCGTACGTCGAGA<br>AGTACTCCGGCGACTACAAGGACCACGACGGGGACTACAAGGACCACGA<br>CATCGACTACAAGGACGACGACGACAAGTCCCGCCTCGAGTCGGGCGAC<br>TACAAGGACCACGACGGGGACTACAAGGACCACGACATCGACTACAAGG<br>ACGACGACGACAAGAGCCGC

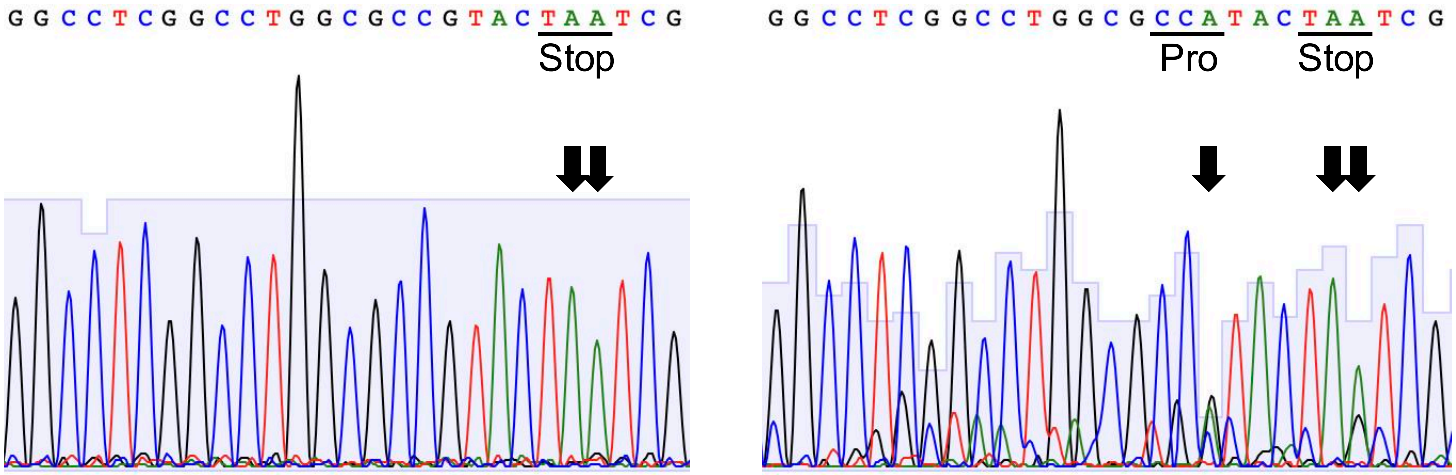

Figure S1
Sanger sequencing chromatograms of the target region with the predicted C to T mutation in the m*actI-ORF2* strains. Mutated bases are indicated by black arrows.

```
SCO3413     1 MSYSVGQVAGFAGVTVRTLHHYDDIGLLVPSERSHAGHRRYSDADLDRLQQILFYRELGF  60
SLA_TipA    1 MGYSVGQVAGFAGVTVRTLHHYDEIGLLSPSGRSGAGHRRYDDADLDRLQRILFYRELGF  60

SCO3413    61 PLDEVAALLDDPAADPRAHLRRQHELLSARIGKLQKMAAAVEQAMEARSMGINLTPEEKF 120
SLA_TipA   61 PLDEVAVLLDDPESDPREHLRRQHALLSDRIARLQQMAKAVEHAMEAKKMGINLTPEERF 120

SCO3413   121 EVFGDFDPDQYEEEVRERWGNTDAYRQSKEKTASYTKEDWQRIQDEADELTRRFVALMDA 180
SLA_TipA  121 EVFGDKDPEQYAEEAERRWGGTEAYAESQRRAAAYTKADWQRIQDEVADWGGRYAALVAA 180

SCO3413   181 GEPADSEGAMDAAEDHRQGIARNHYDCGYEMHTCLGEMYVSDERFTRNIDAAKPGLAAYM 240
SLA_TipA  181 GEPADGEAAMDLAEEHRRHICDRYYECGYEMHVCLGEMYVADERFKAFYDGMGAGVAEHL 240

SCO3413   241 RDAILANAVRHTP                                                253
SLA_TipA  241 RDSITANAVRKA-                                                252
```

Figure S2

Sequence alignment of SCO3413 (TipA) from *S. coelicolor* A3(2) with putative TipA (SLA_TipA) from *S. lavendulae* FRI-5. Identical amino acid residues are highlighted in black.

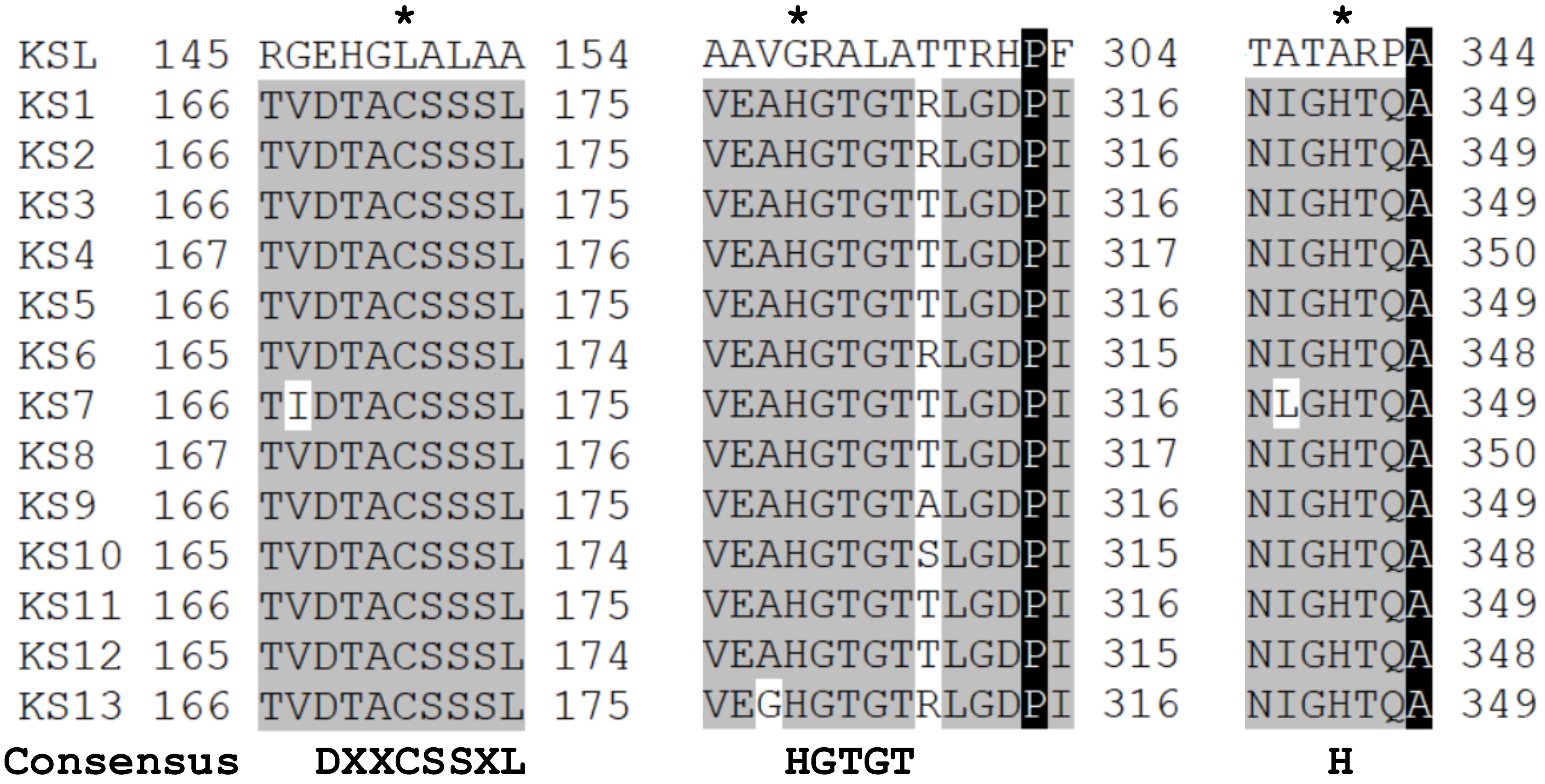

Figure S3

Sequence alignment of the conserved motifs in the KS domain core regions from LadA PKSs. The numbers indicate amino acid positions within each domain. Black and gray boxes in the alignment indicate positions at which the same amino acid is found and at least 70% of the amino acids match, respectively. Asterisks indicate the conserved catalytic triad of C-H-H.

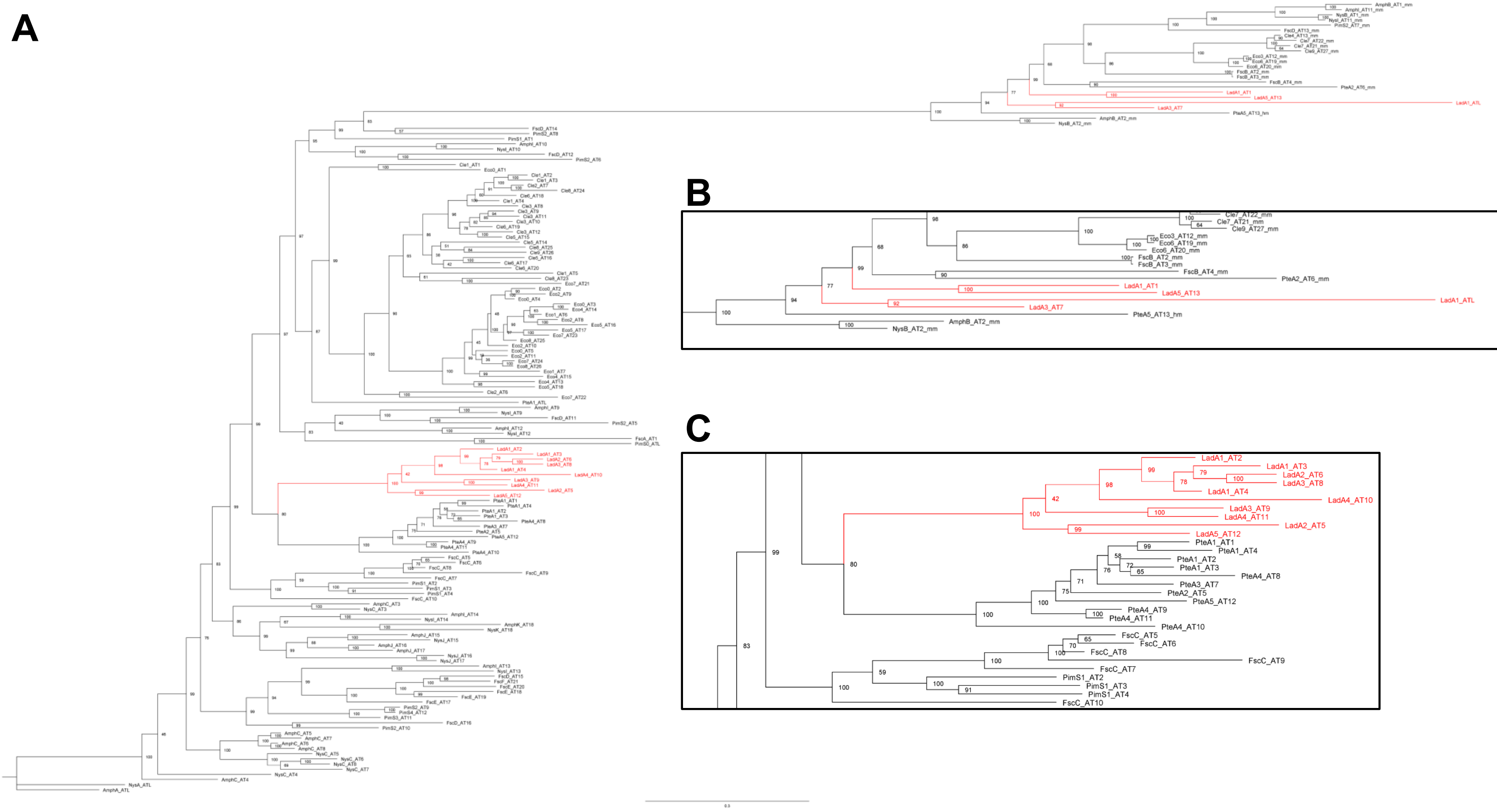

Figure S4

Analysis of the AT domains from the LadA PKSs using a phylogenetic tree. Posterior probabilities are shown at the nodes, and the bar indicates substitutions per site. Subclades colored red indicate AT domains on the lavencidin biosynthesis gene cluster. Amino acid sequences of AT domains were retrieved from the databases DoBISCUIT (https://www.nite.go.jp/nbrc/pks/) and ClusterCAD (https://clustercad.jbei.org/). These amino acid sequences were aligned with Clustal Omega (1.2.2) implemented in Geneious Prime 2023.2.1 (Biomatters Ltd.) with automatically adjusted settings for the number of sequences in refinement iterations. Bayesian phylogeny was conducted by MrBayes (ver. 3.2.7a) on CIPRES Science Gateway (http://www.phylo.org/) with the following parameters: aamodelpr = fixed(LG); ngen = 20 000 000; rateopts = invgamma; nruns = 2: nchains = 4: temp = 0.2: relburnin = yes; burninfrac = 0.25. The substitution model was selected by ModelTest-NG. The phylogenetic tree was rendered with FigTree version 1.4.4 (http://tree.bio.ed.ac.uk/software/figtree/). (A) Overall diagram of the phylogenetic tree of AT domains. (B) Partial diagram of the phylogenetic tree of AT domains including LadA1_ATL, LadA1_AT1, LadA3_AT7, and LadA5_AT13. (C) Partial diagram of the phylogenetic tree of AT domains including LadA1_AT2, LadA1_AT3, LadA1_AT4, LadA2_AT5, LadA2_AT6, LadA3_AT8, LadA3_AT9, LadA4_AT10, LadA4_AT11, and LadA5_AT12.

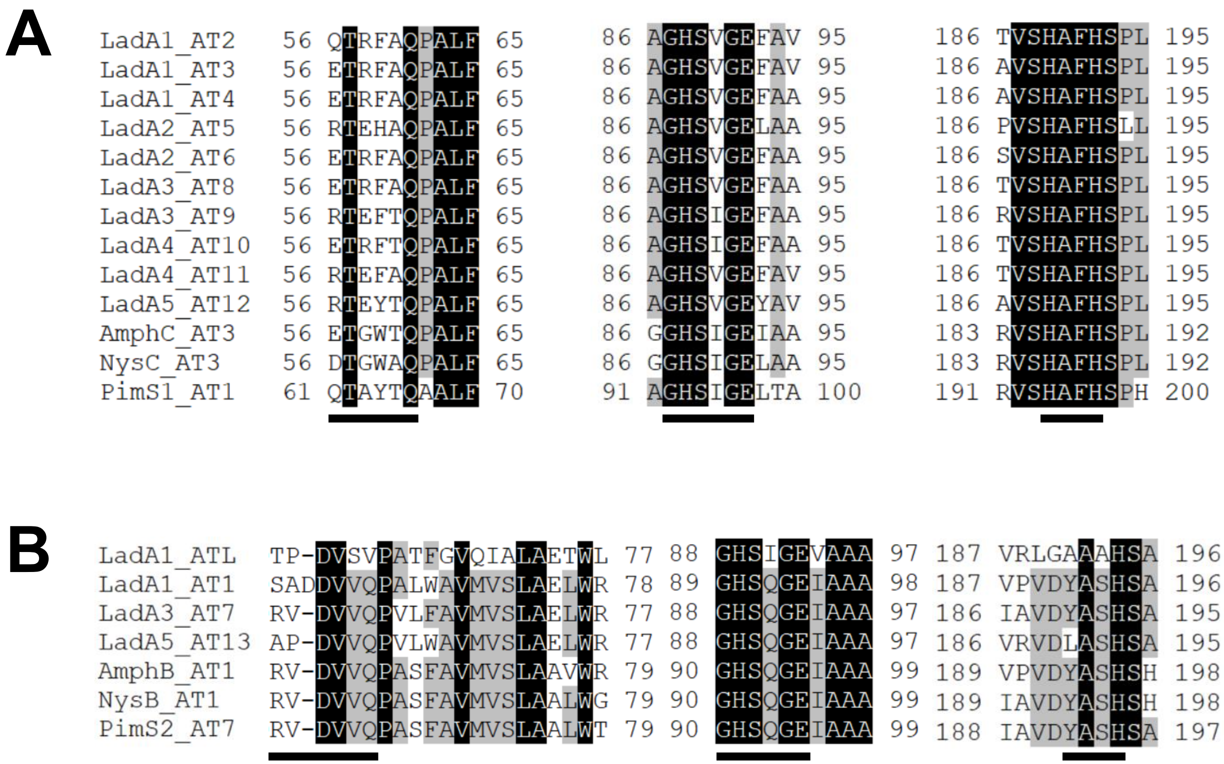

Figure S5

Analysis of the AT domains from LadA PKSs by amino acid sequence alignment. The numbers indicate amino acid positions within each domain. Black and gray boxes in the alignment indicate positions at which the same amino acid is found and at least 70% of the amino acids match, respectively. The AT domains and their corresponding sequence accession numbers are as follows: AmphB/AmphC (AAK73513/AAK73514) for amphotericin in *Streptomyces nodosus* ATCC 14899; NysB/NysC (AVX51107/AVX51108) for nystatin in *Streptomyces noursei* ATCC 11455; PimS1/PimS2 (CAC20931/CAC20921) for pimaricin in *Streptomyces natalensis* ATCC 27448. Three dominant motifs, composed of six, six, and four amino acid residues, are underlined. (A) Sequence alignment of the conserved motifs in AT domains including LadA1_AT2, LadA1_AT3, LadA1_AT4, LadA2_AT5, LadA2_AT6, LadA3_AT8, LadA3_AT9, LadA4_AT10, LadA4_AT11, and LadA5_AT12. (B) Sequence alignment of the conserved motifs in AT domains including LadA1_ATL, LadA1_AT1, LadA3_AT7, and LadA5_AT13.

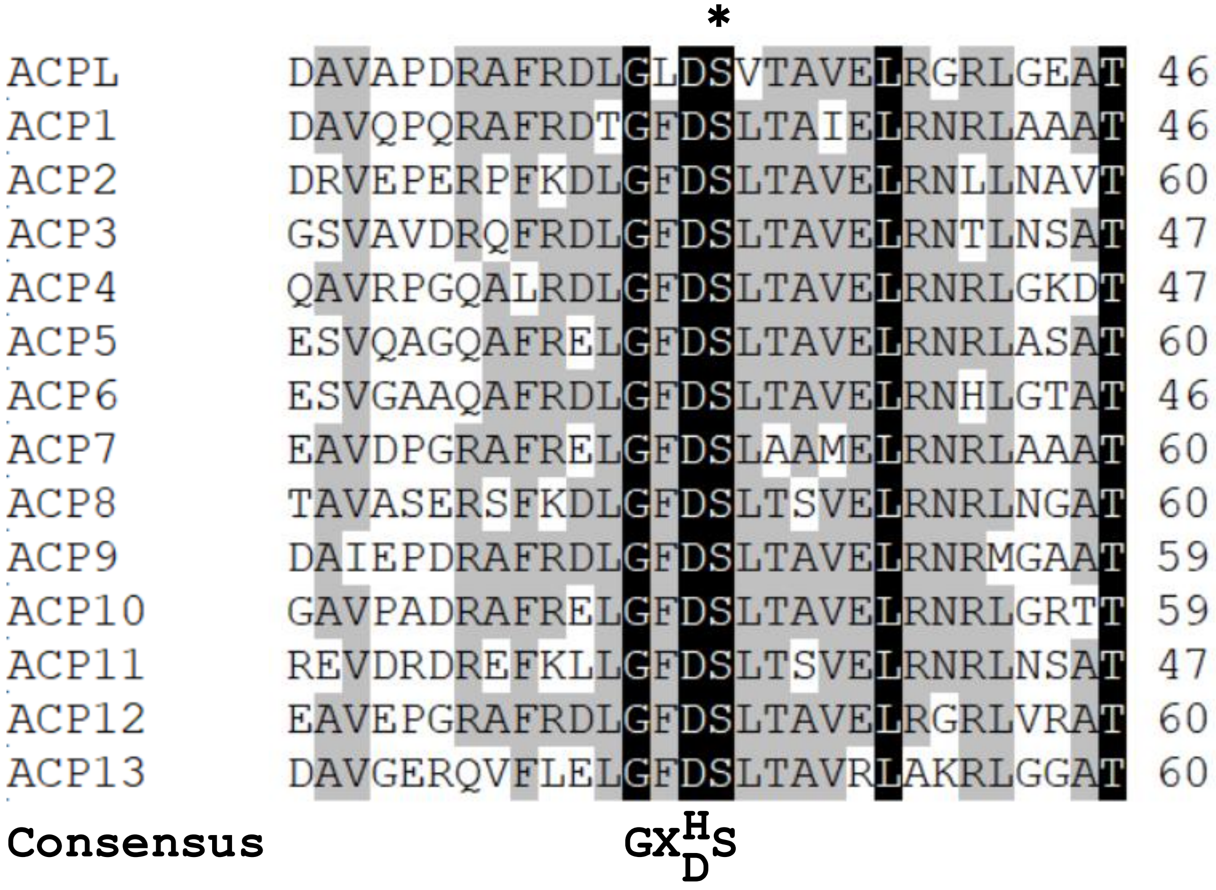

Figure S6

Sequence alignment of the conserved motifs in the ACP domain core regions from LadA PKSs. The numbers indicate amino acid positions within each domain. Black and gray boxes in the alignment indicate positions at which the same amino acid is found and at least 70% of the amino acids match, respectively. An asterisk indicates the active site Ser residue acting as the phosphopantetheine-binding site.

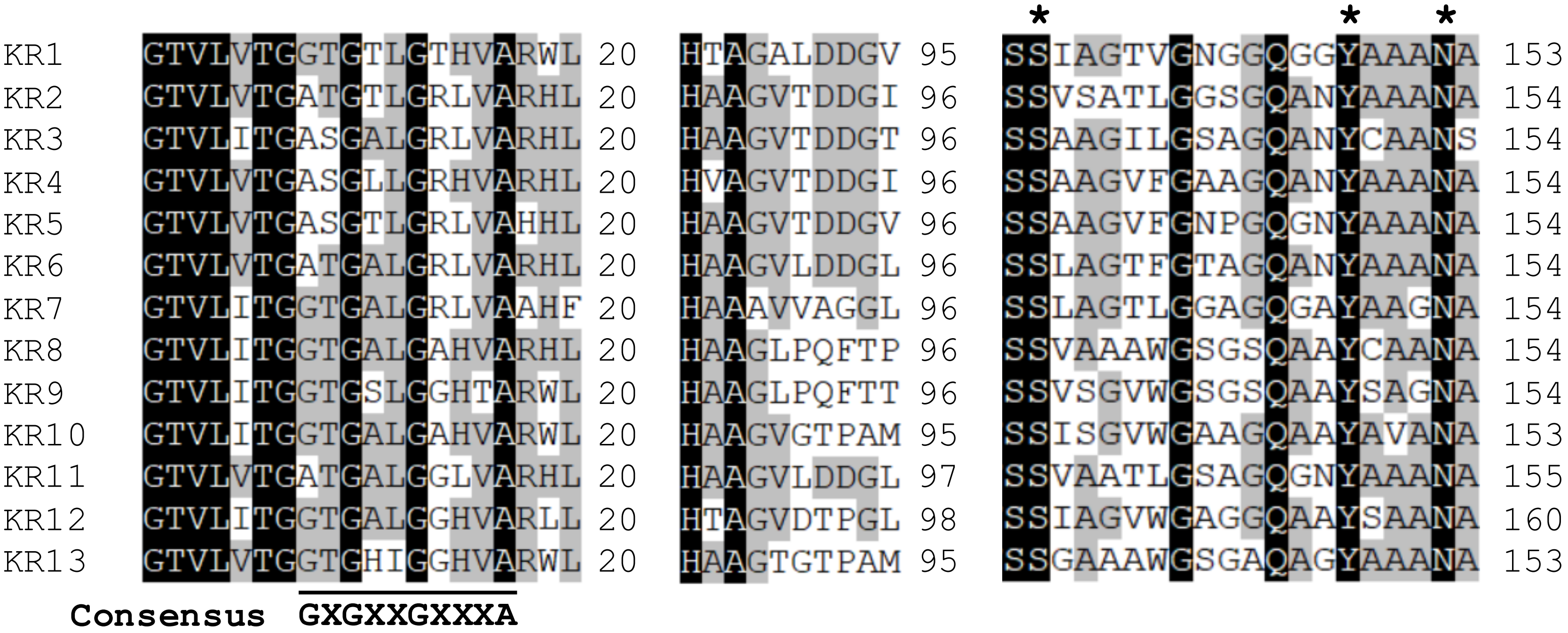

Figure S7

Sequence alignment of the conserved motifs in the KR domain core regions from LadA PKSs. The numbers indicate amino acid positions within each domain. Black and gray boxes in the alignment indicate positions at which the same amino acid is found and at least 70% of the amino acids match, respectively. The consensus sequence for the NADP(H)-binding site is underlined. The catalytic triad of S-Y-N is indicated by asterisks.

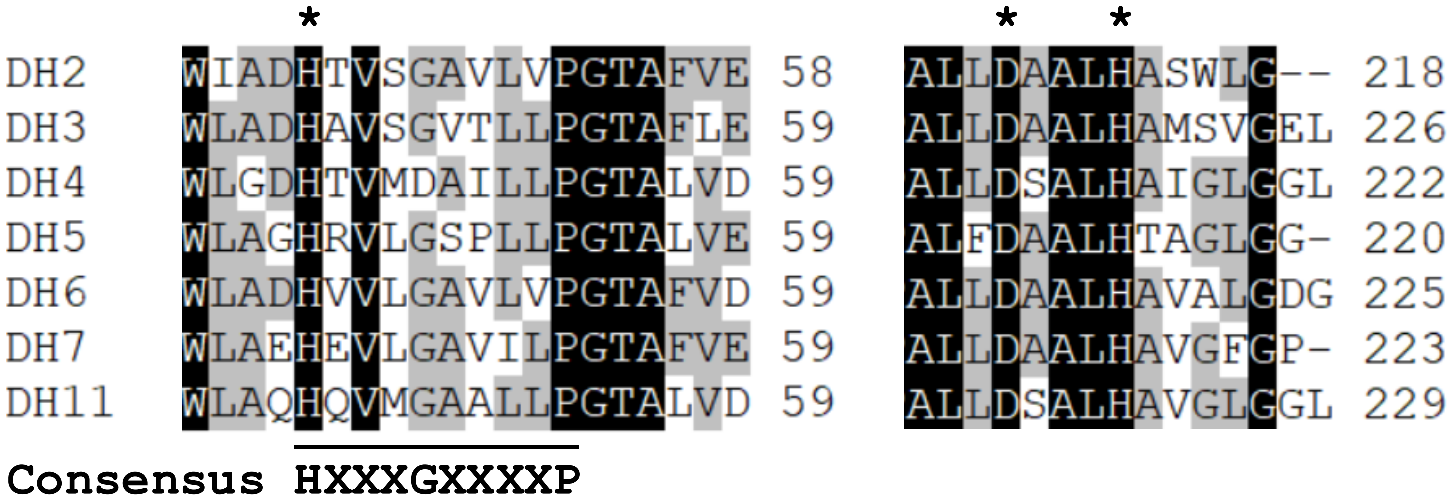

Figure S8

Sequence alignment of the conserved motifs in the DH domain core regions from LadA PKSs. The numbers indicate amino acid positions within each domain. Black and gray boxes in the alignment indicate positions at which the same amino acid is found and at least 70% of the amino acids match, respectively. The conserved HXXXGXXXXP motif for DH domains is underlined. The proposed catalytic His, Asp, and His residues are marked with asterisks.

ER11	LLVHSAAGGVGMAA	144
**Consensus**	**LXHXXXGGVG**	

Figure S9
Sequence alignment of the conserved motif in the ER11 domain core region from LadA4 PKS. The number indicates the amino acid position within the domain. The conserved amino acids in the proposed NADP(H)-binding site LXHXXXGGVG are highlighted in gray.
